# Process evaluation of a community-based program for prevention and control of non-communicable disease in a developing country: The Isfahan Healthy Heart Program, Iran

**DOI:** 10.1186/1471-2458-9-57

**Published:** 2009-02-12

**Authors:** Katayoun Rabiei, Roya Kelishadi, Nizal Sarrafzadegan, Heidar Ali Abedi, Mousa Alavi, Kamal Heidari, Ahmad Bahonar, Maryam Boshtam, Karim Zare, Shahryar Sadeghi

**Affiliations:** 1 Process Evaluation Unit, Isfahan Cardiovascular Research Center, Isfahan University of Medical Sciences, Isfahan, Iran; 2 Pediatric Preventive Cardiology Department, Isfahan Cardiovascular Research Center, Isfahan University of Medical Sciences, Isfahan, Iran; 3 Cardiology Department, Isfahan Cardiovascular Research Center, Isfahan University of Medical Sciences, Isfahan, Iran; 4 Education Development Center, Isfahan University of Medical Sciences, Isfahan, Iran; 5 Nursing Department, Isfahan University of Medical Sciences, Isfahan, Iran; 6 Provincial Health Center, Isfahan University of Medical Sciences, Isfahan, Iran; 7 Health Unit, Isfahan Cardiovascular Research Center, Isfahan University of Medical Sciences, Isfahan, Iran; 8 Laboratory Department, Isfahan Cardiovascular Research Center, Isfahan University of Medical Sciences, Isfahan, Iran; 9 Occupational Health of Provincial Health Center, Isfahan University of Medical Sciences, Isfahan, Iran

## Abstract

**Background:**

Cardiovascular diseases are the most common cause of mortality in Iran. A six-year, comprehensive, integrated community-based demonstration study entitled Isfahan Healthy Heart Program (IHHP) conducted in Iran, and it started in 2000. Evaluation and monitoring are integrated parts of this quasi-experimental trial, and consists of process, as well as short and long-term impact evaluations. This paper presents the design of the "process evaluation" for IHHP, and the results pertaining to some interventional strategies that were implemented in workplaces

**Methods:**

The process evaluation addresses the internal validity of IHHP by ascertaining the degree to which the program was implemented as intended. The IHHP process evaluation is a triangulated study conducted for all interventions at their respective venues. All interventional activities are monitored to determine why and how some are successful and sustainable, to identify mechanisms as well as barriers and facilitators of implementation.

**Results:**

The results suggest that factory workers and managers are satisfied with the interventions. In the current study, success was mainly shaped by the organizational readiness and timing of the implementation. Integrating most of activities of the project to the existing ongoing activities of public health officers in worksites is suggested to be the most effective means of implementation of the health promoting activities in workplaces.

**Conclusion:**

The results of our experience may help other developing countries to plan for similar interventions.

## Background

Cardiovascular diseases (CVD) are the leading cause of mortality worldwide [1]. It is well documented that unhealthy lifestyle may account for as much as 50% of CVD-related mortalities [2]. Lifestyle modification has long been considered essential in curbing non-communicable diseases notably cardiovascular diseases [3]. However, the efficacy of such interventions in developing countries is less clear and data from interventional studies in such countries are limited. Moreover, there is paucity of interventional studies targeting the whole community; most have selected specific groups rather than the whole community. In 1995, circulatory diseases, mainly CVD, accounted for 47.3% of all Iranian deaths, with a higher prevalence among people of lower socio-economic status [4]. As a public health response to the high prevalence of CVDs in Iran, a six-year, action oriented, comprehensive and integrated community-based study, entitled Isfahan Healthy Heart Program (IHHP), was designed and launched in 2000. This program provided an opportunity to assess whether lifestyle interventions are effective or not in developing countries. The program targeted individual community and environmental changes to support health behavior modification. IHHP consists of three phases. The situation analysis (Phase I) of the program was conducted in the cities of Isfahan and Najafabad (cities of intervention) and Arak (as the reference area). Phase II consists of a 5-year interventional program. Phase III will be conducted in both intervention and reference areas to evaluate program outcomes [5].

The interventional program targeted the general population as well as specific groups in urban and rural areas. IHHP strategies have integrated activities covering different fields – health promotion, disease prevention, healthcare treatment and rehabilitation. Key strategies for intervention activities included public education through mass media, inter-sectoral collaboration, professional education and involvement, marketing and organizational development, legislation and coordination, policy development as well as research and evaluation.

IHHP is focused on healthy nutrition, increased physical activity, tobacco control and stress management. Interventions targeted individuals, populations and the environment based on the results obtained from the baseline surveys, needs assessment, as well as existing health services. The program comprised 10 distinct projects each targeting different groups, including Women's Healthy Heart Project, Heart Health Promotion from Childhood, Health Professional Education Project, Youth Healthy Heart Project, Worksite Intervention Project, Healthy Lifestyles for High Risk Groups, Healthy Food for Healthy Communities, Isfahan Exercise Project, Non Governmental Organizations (NGOs) and Volunteer Intervention Project and finally, Healthy Lifestyle for Cardiac Patients.

Each project is supervised by a steering committee of directors that includes academics, public health officers, stakeholders and policy makers. All directors are members of the High Council of IHHP and are involved in planning, implementing and evaluating their projects. Directors of different interventions work intensively and closely with representatives of mass media (television, newspapers, radio, etc.), health professionals (administrators, physicians, nurses, health workers and volunteers, social workers, school staffs, etc.), business and market leaders (food-industry, groceries, bakeries, fast food shops), staffs of the key NGOs and local political decision makers (county, municipal and provincial leaders. Given that a community-based program of this scale has no precedent in developing nations, the program was supported by the World Health Organization (WHO) as a model for such countries.

As we described previously, the IHHP evaluation consists of process, impact and outcome evaluation. The process evaluation (PE) is of fundamental importance in the IHHP [6]. PE is critical to modification and improvement of intervention strategies. Nonetheless, it has barely been given high priority in similar studies thus far conducted [7-10]. It usually addresses the internal validity of an intervention by ascertaining the degree to which the program was implemented as designed or intended. PE is recommended for use in either new or revised interventions, as a means to understand the specific components of the program being delivered [11-13]. This type of evaluation employs quantitative and qualitative methods to assess an intervention, *i.e*, the number and types of activities and the consistency of interventions with their objectives. In addition to providing an overall understanding of program implementation, PE is used for the identification of major obstacles that can affect the program services and the quality of implementation [14]. This paper presents the design of PE in IHHP, and the PE results for some IHHP strategies. Given the large amount of data concerning the PE of the ten interventional projects of the IHHP, here we present the results of the PE of only one project, the Worksite Intervention Project. Strategies, data collection methods, target community and evaluation indicators of the process evaluation of this project are summarized in Table 1.

**Table 1:** Strategies, data collection methods, target community and evaluation indicators of the process evaluation of the "Worksite Intervention Project-IHHP"

**Strategy**	**Data collection method**	**Target community**	**Evaluation indicator**
1 – Improving nutrition in factory restaurants	Checklists	Workers and staff members	Number of restaurants where nutrition has been improved
2 – Providing education on cardiovascular risk factors	Questionnaires	Workers and staff members	Number of individuals receiving education
3 – Reducing smoking in offices	Interviews Questionnaires	Workers and staff members	Number of venues where antismoking regulations have been enforced
4 – Improving physical activity in workplace	Questionnaires	Workers and staff members	The number of individuals with increased leisure time and/or transportation activities

To design the project interventions, data relating to behavior, attitude, skills and knowledge (BASK) of employees towards nutrition, smoking, and physical activities, as well as the prevalence of CVDs and their related risk factors in the year 2000 were taken from the baseline survey. The interventions were then designed by identifying stakeholders, existing resources, and assessing feasibility of implementation. This project was designed to promote the lifestyles of employees of offices and factories. The interventional activities of this project consist of:

• training occupational physicians, health assistants and health volunteers who will in turn train other factory workers;

• introducing dietary modifications into workplace restaurants;

• enforcing no-smoking regulations in workplaces;

• Using the existing screening system for detecting CVD risk factors to identify at-risk employees and workers, and increasing physical activity at work.

## Methods

The IHHP process evaluation is a triangulated study. Triangulation is the combined use of two or more theories, methods, data resources, investigators or analysis methods in the study of the same phenomenon. In the present study, qualitative as well as quantitative methods were combined in order to acquire rich data and also increase the overall validity of the study. Triangulation was conducted for all interventions. All interventional activities were monitored to determine why and how some are successful and sustainable, to identify mechanisms, barriers and facilitators of implementation as well as the possibility of integration and dissemination of activities based on the results obtained from monitoring and evaluation.

The evaluation process of IHHP can be summarized as follows:

1. Establishment of a process evaluation committee

2. Designing questionnaires that can identify the interventions

3. Completion of the questionnaires by respective project managers

4. Designing questionnaires for implementing the interventions according to the target population and places of intervention

5. Determination of the individuals targeted by each intervention

6. Simple random sampling of the target population

7. Completing the questionnaires

8. Data entry

9. Analysis of the completed questionnaires

10. Qualitative studies, including interviews and focus group discussion (FGD)

11. Collecting the results of qualitative studies

12. Drawing general conclusions from the triangular study

13. Providing the feedback to project managers

14. Continuing, improving, or dropping the interventions, or designing a new intervention in view of the feedback provided by project managers

15. Notifying the process evaluation committee of the changes

16. Back to number 3

Data were collected by questionnaires, individual interviews, and focus group discussions. First, we carried out quantitative studies in order to draw the qualitative study guideline. Taken into account that qualitative research is conducted on people aware of the topics related to the study, and not on random samples, we selected purposive participants and specific initial questions in the qualitative part of the study based on findings resulted from the quantitative part. They were selected based on the type of the intervention and the target groups of each activity. The questions consisted of the possible facilitators and barriers of activities, as well as the extent of the intervention received by the target group. Initially, evaluation committees consisting of principal project managers and external evaluators,(i.e., individuals who were not involved in the project and were selected to conuct the quality control of the study)were assembled. In order to obtain information about the implementation of the ten interventional projects of IHHP, and the application of community principles, the PE was conducted by an independent team of researchers consisting of health professionals not directly involved in conducting the project. The committee designed a questionnaire according to the project interventions. The questionnaire addressed questions to assess the interventions and to see whether they have achieved the established objectives according to the timetable of activities, existing resources (including human resources, budget and various facilities), the points of views of stakeholders, obstacles confronted by project managers and the extent of integration of projects into the existing systems. The questionnaires included both open-ended and closed-ended structures, and were completed by managers of individual projects.

Data on implementation of the IHHP, exposure to interventions, diffusion of intervention activities, as well as the process of changing health behavior and risk factors were obtained in the annual behavioral survey, annual process notes collected by the related health center units as part of their routine monitoring in intervention venues, as well as during site visits. In addition, checklists for monitoring of activities were completed at regular intervals. In the questionnaires, each project manager provided a thorough outline of the respective project. All input information (types of interventions, human resources, and the amount of the budget spent) and output information (number of intervention venues, number of trained individuals, number of approved legislations, the project managers' impression of the success of interventions, feasibility of integrating the projects into the existing system, impediments and facilities) were collected.

The evaluation and monitoring questionnaires were designed based on questionnaires completed by project managers. The objective was to gauge the extent of interventions, their success, effectiveness, sustainability and the possibility of integration into health programs. The questionnaires were designed according to the goals of interventions, type of intervention (educational, environmental, or legislative), target population (children, women, employees, patients, public health personnel, youth, NGO members and other volunteers, and the community at large, especially as regards nutritional interventions in food preparation and distribution centers) and places of intervention (schools, kindergartens, offices, factories, hospitals, health and treatment centers, restaurants, bakeries, cafeterias, etc.). A separate questionnaire was designed for each intervention based on the above criteria. Fourteen questionnaires were designed for ten interventional projects. The questionnaires consisted of two distinct parts: 1) questions addressed to managers and intermediary target populations such as health volunteers in offices, factories, schools, kindergartens, and other places of intervention, and 2) questions addressed to the target population. The interviewees' knowledge of the program, extent of their involvement in the interventions, their degree of satisfaction with the program, and the impact they felt the interventions made on their lifestyle were assessed by these questionnaires. The questionnaires included questions with three choices (e.g., *I agree, I have no idea, I disagree *or *Yes, I don't know, No*), which aimed to evaluate the reach of interventions in their intended venues and to determine the success of the interventions and the feasibility of continuing them.

A simple random sample of the target community undergoing the interventions (based on information supplied by project managers) was interviewed and the questionnaires were filled by interviewers. Sample size in the intervention venues of each project was calculated at fifty. The project managers were requested to present to the evaluation committee with all of the circulars issued by stakeholding centers in line with interventions. The evaluation committee was also informed of the number of venues where interventions were implemented. With the project managers' viewpoints regarding the places of intervention, the interviewees were selected from the individuals targeted by the interventions. The number of interviewees varied between 50 for employees and 500 for students, according to the objectives of the interventions and their target populations. These individuals were selected from the community of intervention and the questionnaires were completed by trained interviewers. Moreover, given that the implementation of each intervention required meetings with policymakers and stakeholders, as well as the intermediary and target populations, the minutes of meetings corresponding to the interventions were collected. Also, as the interventions were adopted as legislations by organizations and provincial and city administrations, the project managers were requested to collect all the related official directives issued by stake-holding organizations and present them to the evaluation committee. The responses to the close-ended questions of the final survey were entered directly into computer files for statistical analysis. The open-ended questions were coded using classical content analysis procedures, and then transferred to computer files. Data were analyzed by SPSS (SPSS, Chicago, Inc). The data are used in a descriptive manner to illustrate rather than test findings.

After completing the questionnaires and the quantitative study, we began the qualitative study. It was conducted based on the data obtained from the questionnaires and individual interviews, and in some cases by focus group discussions. The interviewers were individuals from outside IHHP who had experience on similar interviews in other research studies, and who became familiar with IHHP by attending a number of sessions. They consisted of health professionals and or people working in different organizations related to the activities of various interventions. Necessary information was given by face to face education, pamphlets and video films. The first interviews were conducted with the project managers to make sure about the expertise of the interviewers. Initially, they conducted complete interviews with project managers, inquiring about their impressions of the programs, methods of implementing the programs and the experience they gained during the process of implementation, as well as their views regarding the prerequisites of integrating the programs into the existing system. Interviews were then conducted at the project managers' discretion with stakeholders, project colleagues and organizations with experience in performing the interventions. On average, each interview lasted between 45 and 60 minutes. The interviews were conducted in an extrapolative manner and the interviewees were asked general questions. During the interviews, no clues or directions were provided by the interviewers and only when the interviewees seemed to digress, they were led back to the question at hand. All the interviews were recorded after obtaining the interviewees' oral consent. After each interview, the written transcript was prepared and reviewed several times by the interviewer and all the sub-concepts and bits of meanings were extracted. The perceived meanings were classified into more generalized concepts and the process continued until basic concepts were extrapolated. The materials were reassessed by a qualitative analyst (a faculty member of Isfahan University of Medical Sciences with good expertise in qualitative research at national level) and the same process was applied until extrapolation of basic concepts was complete. For each project, the interviews continued until a level of information saturation was reached. On average, six interviews were conducted for each project. After analyzing the questionnaires, we performed qualitative studies to determine the reasons for success or failure of interventions, obstacles to interventions, and other concepts expected of qualitative studies.

Discussing all the results of process evaluation is beyond the scope of the present study; hence we provide a single example. As earlier indicated, the interventions designed within the worksite project were based on the results of phase I of IHHP in coordination with related policymakers and stakeholders. Based on design, all factories and offices in the cities of Isfahan and Najafabad (104 offices, 121 factories, 14565 workers and employees) were targeted by this project. Similar to process evaluation for other interventions of IHHP, the questionnaire shown in Table 2 was initially completed by project managers. Results showed that the projects consisted of four main interventions aimed at improving the lifestyles of employees and reducing the prevalence of cardiovascular diseases in them. The interventions were initially performed in centers volunteering to cooperate with the program. A physician plus a public health officer or a health care volunteer at each center or health system were designated as project assistants; they were referred to as "project colleagues" in the questionnaire. In addition, the project managers specified the offices and factories where the interventions were conducted and described the type of interventions. To conduct process evaluation, a questionnaire was designed to assess the extent of implementation of the intervention, its effects on the target population, and satisfaction of the target population with the intervention. The questionnaires were completed by fifty individuals in factories and/or offices of intervention, selected by simple random method. The questionnaires were completed by public health officers who normally perform surveillance of health indicators in worksites on behalf of the public health centers. The checklists for monitoring intervention activities were also completed by public health officers collaborating with the committee, in all offices and factories of intervention.

**Table 2:** Sample of the questionnaires used in the process evaluation of IHHP interventional projects

**Project title**
**The personnel involved in implementing the project**

Principal project managers (Please specify the names of all project managers, their workplace and contact number)

Principal project colleagues (Please specify the names of all project managers, their workplace and contact number)

Other individuals involved in project implementation (Please specify the names of all project managers, their workplace and contact number)

Title of the stakeholding organization (Please specify the name of the volunteer in the organization, address and contact number)

**Goals and objectives**

Primary project goal

Secondary objectives

**Interventions**

Title of intervention

1. Project manager

2. Project colleagues

3. Other individuals involved in project implementation

4. Organizations cooperating with the project

5. Goals pursued by intervention

6. Target community

7. Type of intervention

8. Venues of intervention (Please specify all intervention venues, names of volunteers, addresses and contact numbers)

9. Date of starting interventions

10. Date of ending interventions (if applicable)

11. Amount of spent budget (direct and indirect budgets), Source of budget

12. a) Method of education (class, face-to-face, educational materials, gatherings, education via media, etc.)

12. b) Please specify in detail the type and method of implementing your non-educational interventions (if applicable)

13. Challenges confronting the implementation of interventions

14. How do you rate the success of intervention?

15. Given the explanations, has the intervention been modified or totally discontinued?

16. State your degree of satisfaction with this intervention.

17. Do you think this intervention must be continued, modified, or discontinued? State your reasons.

18. Have the goals of your respective intervention been stabilized in the system?

19. How do you assess the future continuity of habits encouraged by your respective intervention?

20. Do you think this intervention can be nationalized? Please state your reasons.

After completion of monitoring checklists and evaluation questionnaires, a qualitative study was performed to determine the reasons for success or failure of interventions, as well as obstacles and impediments. Ten participants in the project were interviewed including office administrators, factory managers or their representatives as project colleagues (physicians, public health officers or office/factory health care volunteers) and individuals in the target population. The interviews were continued until data saturation was reached. Ten individuals were interviewed for a total of nearly 12 hours. The interviews were recorded and transcribed. Primary and secondary coding was conducted and the concepts were extracted. Ultimately, the results were provided to project managers to improve the interventions.

## Results

At the beginning of the program in 2000, 10 factories and 50 offices agreed to the implement the interventions in their respective worksites. The program was implemented in 45% of worksites by the end of 2005 when 176 public health officers from the worksites worked with the project. During the 4 years of interventions, 102 educational sessions were organized in different factories. The employees were trained through 12 educational seminars that were integrated to the regular health educational classes of workers/employees organized by the Provincial Health Center. The interventions of this project included risk factor education, lifestyle modification, improvement of cooking methods in restaurants and integration of physical activity in daily routines. The quantitative questionnaires and monitoring checklists were completed in the target community, randomly included in the interventions. Data analysis of monitoring checklists of interventions aimed at nutritional improvement in restaurants of factories demonstrated decreased consumptions of salt, replacement of hydrogenated fat with oil, consumption of yogurt and salads three times per week, cooking chicken in boiling water rather than frying in oil/fat and no more delivery of soft drink (soda) in 40% of these restaurants. Data analysis of the questionnaires revealed that 39.5% of the target community received education. Their opinions regarding the educational programs are presented in Tables 3, 4, 5.

**Table 3:** Results of the process evaluation of some interventions of the IHHP-Worksite Intervention Project

**Title of intervention**	**Holding gatherings**		**Face-to-face education**		**Class education**		
	Date/frequency of gatherings	Yes/No/Number of participants	Number of individuals receiving education	**Education venue**	**Number of teachers**	**Number of health workers**	**Number and frequency of classes**
Risk factor education	-	Yes/76	95	Industrial units covered by intervention	7	176	seasonal
Nutrition education	-	-	-	Factories and companies	-	-	Variable
Nutrition education	-	-	46	Companies	4	112	Four classes every six months
Nutrition education	-	-	130	Companies	3	60	Five classes every three months
Education on physical activity	-	Yes/80	23 directors, 55 health experts and volunteers	Industrial units where interventions are implemented	-	-	-

**Table 4:** Results of PE questionnaires of educational interventions in worksites

**Frequency (%)**		
**Types of education**		
Class	50	25
Poster	62	31
Pamphlet	22	13
Seminars	11	6
		
**Subjects of education**		
Nutrition	72	**36**
Exercise	64	**32**
Stress	42	**21**
Risk factors	76	**38**
		
**Satisfaction of Education**		
Yes	64	32
No	18	9
No idea	18	9
		
**Attraction of education**		
Yes	72	36
No	4	2
No idea	24	12
		
**Increase in knowledge**		
Yes	90	**45**
No	4	**2**
No idea	6	**3**
**Change of practice**		
Yes	86	43
No	8	4
No idea	6	3
		
**Was the method of education appropriate?**		
Yes	78	39
No	14	7
No idea	8	4

**Table 5:** Process evaluation of interventions aimed at improving physical activity and preventing smoking in workplaces

**Personnel**	**Yes(%)**	**No(%)**
**Are there exercise hours at your office?**		

Before implementation	53.6	42.9
After implementation	60.7	32.1
**Do you perform stretching exercises during work?**		
Before	35	60.7
After	39.3	57.1
**Do you benefit from the sport budget of the factory?**		
Before	28.6	67.9
After	32.1	60.7
**How to you travel to and from work?**		
**Before**		
Public bus service	3.6	
On foot	3.6	
Bicycle	7.1	
Private car	14.3	
Office bus service	7.4	
**After**		
Public bus service	5.6	
On foot	3.6	
Bicycle	7.1	
Private car	14.3	
Office bus service	6.7	
**Are antismoking regulations enforced at your workplace?**		
Before	60.7	35.7
After	71.4	10.4
**Do you smoke at workplace?**		
Before	28.6	67.9
After	28.6	35.7
For fun	35.7	

Only 57% of the factories could improve their food menus within their existing means. These improvements included substituting hydrogenated fat with liquid oil, reducing red meat consumption and increasing the consumption of fish and vegetables. Nearly 50% of workers expressed satisfaction with the type and taste of healthy foods in their factory restaurant. On this note, mention can be made of the remarkable success of one of the factories in establishing a "Healthy Heart Restaurant". The restaurant, originally built to offer meals to 70 out of the 7000 workers, and has been expanded to cater for 1200, is currently unable to meet all demand for healthy food due to inadequate personnel, equipment and space

**Figure 1:**
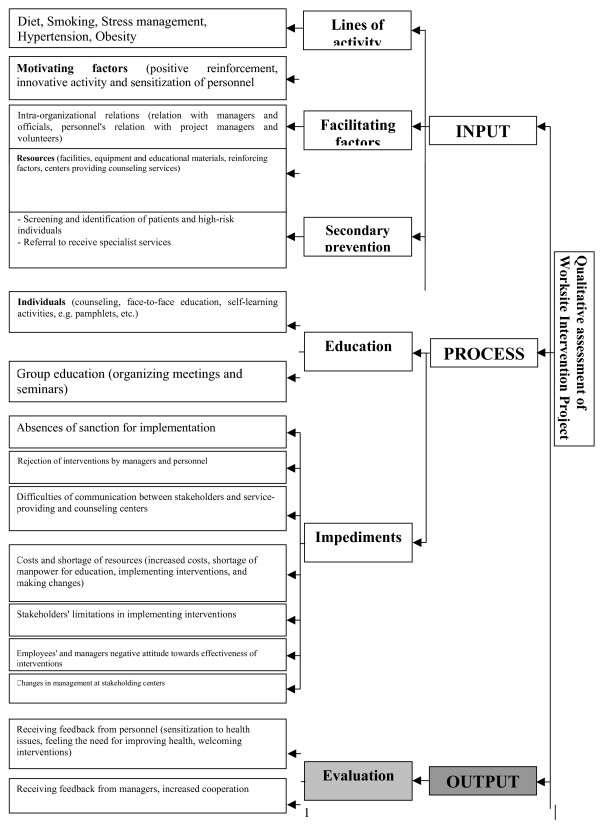
**Qualitative assessment of Worksite Intervention Project**.

A qualitative study in the form of interviews was conducted after completion of questionnaires and monitoring checklists (Figure 1). The results of this study can be divided into two categories, namely contributing and impeding factors. Based on qualitative evaluation conducted using information collected from factory managers and public health officers, the following results were obtained concerning the factors impeding or facilitating the project.

"*... We were initially faced with resistance; they did not accept the changes, however, following what they saw in practice, they gradually declared their willing towards accepting the prevention services ...*" (participant no. 4).

### 1. Facilitating factors

In the present study, our experience with concepts like *motivating factors*, *relationships *and *resources *bear testimony to the facilitating factors.

#### 1-1. Motivating factors

##### 1-1.a. Positive reinforcement

This sub-concept suggests that public health officers and project managers have succeeded in motivating their target community to accept and continue healthy behaviors via providing them with positive feedback in response to their healthy behaviors.

##### 1-1.b. Behavioral activities

These activities were also adopted to increase acceptance and stabilize healthy behaviors: "... *We arranged special programs such as the caricature contest for the employees, their children and families to engage them ...in anti-smoking campaigns ... *" (participant no. 35).

##### 1-1.c. Sensitization of workers and employees

As can be inferred from the interviews, after gaining awareness of the positive outcomes of healthy behaviors and the consequences of unhealthy behaviors, the workers and employees make stronger efforts to adopt them:

"*... Today, things are so changed that if the personnel are not served vegetables or dairies once or twice a week, they start complaining to the restaurant health authority...*" (participant no. 3).

#### 1-2. Relationships

##### 1-2-a. Intra-organizational relationships

The study findings showed that correct implementation, as well as follow-up of implementation of interventions at worksites occurs within a host of formal and informal intra-organizational relationships. In this process, the factory public health officers engaged the factory managers and relevant officials in the implementation process of interventions and receive their feedback through written correspondence, or by holding meetings. The relations between the employees and the public health officers at worksites form another aspect of intra-organizational relationships.

"*... Things were done through coordination and cooperation between the management, factory public health officer, and restaurant manager ... *" (participant no. 3).

##### 1-2-b. Inter-organizational relationships

One of the public health officers stated: "*... our colleagues worked hard in different areas and used the consultation and cooperation of the organizations that I mentioned earlier; i.e., the district health centers, the provincial health center, and Isfahan Cardiovascular Research Center ...*" (participant no. 2).

#### 1-3. Resources

The resources included all opportunities, physical facilities and information, which helped drive the project towards its objectives. Otherwise stated, resources are the supporting pillars of interventions and included the following sub-concepts in the current study:

##### 1-3-a. Facilities

"*... We offered them free-of-charge facilities like sneakers, exercise clothes ... paid for by the management, so they would continue to exercise ... *" Said one of the public health officers (participant no. 3).

##### 1-3-b. Educational materials and equipment

*"... The public health officers distributed free educational pamphlets, brochures, newsletters and journals at worksite places. These materials satisfied the workers and increased the employees awareness. ... and this was done in many places ... some brochures and journals were already made at the Cardiovascular Research Center. ... Distribution of newsletter and stuff like that has made a difference ... " *Said one of the public health officers (participant no. 1).

##### 1-3-c. Reinforcements

Reinforcements include opportunities and objectives already in place at stakeholding centers, regardless of project interventions. These factors have exerted a reinforcing effect on project interventions:

"*... The non-smoking program has been made compulsory. Things have so changed that smoking is forbidden in office environments with roofed spaces. This has significantly reduced smoking. ...*" (participant no.1).

##### 1-3-d. Counseling centers

The importance of centers offering counseling is evident from the participants' statements:

"*... Provincial health center, the labor and social affairs office, and the Cardiovascular Research Center, which we started to work with 5–6 years ago gave us good guidance and counseling ...*" (participant no. 3).

### 2. Obstacles to interventions

Study findings suggested that several shortcomings and inhibiting factors impeded the implementation of interventions and achievement of project objectives.

#### 2-1. Refusing to accept the programs

To achieve their goals, programs require changes which may affect the environment, the costs, or even the individuals. The findings of this study indicated that the refusal of the target communities (managers and employees) to accept the programs hinders the implementation of interventions.

##### 2-1.a. Management's refusal to accept the programs

The following statement supports our findings:

"*... The management should be briefed. Then, they'll be able to follow the issue more easily ... *" (participant no. 1).

##### 2-1-b. Employees' refusal to accept the programs

The following statement supports our findings:

"*... We were initially faced with resistance; the employees did not accept the changes. Luckily, following our recommendations and what they saw in practice, they gradually became inclined towards accepting the prevention services ...*" (participant no. 4).

##### 2-1.c. Difficulties in communication between stakeholders and institutes providing counseling

The interaction between stakeholders and institutions providing counseling and other services can be compared to a framework within which the interventions are defined and followed up. The experiences of the participants in the project at hand are suggestive of such hurdles: "*... We expect these centers not to work for profit since any of the personnel who has problems will be referred to the specialists of these centers ...*" (participant no. 3).

##### 2-1-d. Costs and shortage of facilities

As earlier noted, bringing about change at the stakeholding centers requires spending money, which is often met with resistance from the management, occasionally putting the continuation of interventions in serious jeopardy.

##### 2-1-e. Costs

Conducting screening tests for high-risk individuals imposes significant costs.

"*...As the health insurance companies do not cover the costs of some of the tests, this is not workable in many places; I mean employees cannot pay for the tests by themselves ... *" (participant no. 1).

##### 2-1-f. Shortage of human resources

Education, implementing interventions and changes need a number of staff. Shortage of such human resources was mentioned as an important obstacle to the interventions.

##### 2-1.g. Centers' limitations in implementing programs (skills, space, equipments)

Following is how one participant portrayed the limitations: "*... We (restaurant managers) set limits on the number of guests (coming to the healthy heart restaurant) since there's no more room in our saloon ...*" (participant no. 5).

#### 2-2. Negative attitudes

To be implemented, every program needs to create positive feelings towards its outcomes. As found in the current study, the presence of such inhibiting factors has concerned the management and employees about the prospect of the program and its outcomes.

##### 2-2-a. Management's negative attitude towards the effectiveness of the programs

The following statement supports this finding: "*... One of the obstacles was that some employers refused to cooperate, and that's because factory affairs are tied to economic matters ... *"(participant no. 2).

##### 2-2-b. Personnel's negative attitude towards the effectiveness of the programs

There were some negative attitudes about the effectiveness of the interventions, as stated by one of the workers: "*... Many employees thought preventive programs had nothing to do with workplace. But, when we explained things for them, they began to understand ...*" (participant no. 2).

#### 2.3. Changes in management

The participants believed that replacement of managers and the subsequent changes at the stakeholding centers and institutions often stall the programs, resulting in loss of opportunities and working power:

#### 2.4. Lack of sanctions

Successful implementation of every project depends on the subjects' degree of commitment to comply. Absence of sanctions slows down the implementation of programs at different levels. IHHP interventions were also hampered by the absence of sanctions: "*... Implementation of any action should be backed by a sanction. I mean, when we give them a questionnaire or a checklist every month to complete and return, something should oblige them to do so, and someone should follow it up ...*" (participant no. 1).

The quantitative questionnaires were also completed by the target community, randomly included in the interventions.

## Discussion

The current process evaluation was designed to assess the trend of interventions, gauge satisfaction levels, identify problems, impediments and facilitators, and determine the continuity of interventions. In this study, the degree of satisfaction of the target community with the interventions was assessed in addition to the evaluation of the interventions and stakeholders. The results are suggestive of the complete satisfactions of the factory managers with the interventions. Worksite interventions included risk factor education, nutrition improvement at restaurants, capacity building and passing legislations on physical activity and non-smoking. PE aimed to make the most efficient use of available resources in conducting interventions. The main purpose of PE will only be served if the project interventions can be integrated into the target systems based on evaluation results. Interviews, qualitative studies, returned questionnaires and checklists should be used to gauge the success of interventions, as well as the satisfaction of stakeholders and the target community, and accordingly develop strategies towards sustainability and continuity of interventions in the existing health system. Integration of interventions must be pursued in view of the limits of the available resources, and the requirements of the existing policies. Given the rigid nature of organizational budgets, projects requiring additional financial or human resources will ultimately fail.

Understanding the governing policies and priorities of the stakeholders was crucial to the success of interventions. Identifying the available resources and understanding the strengths and weaknesses of interventions and impediments to their implementation constituted yet other important aspects of process evaluation, which could help project managers improve the interventions. This task was accomplished by the Evaluation Committee through conducting interviews and qualitative studies.

Fulfilling the aims of interventions and their overall improvement depends on the feedback information received by project managers. Using this information, project managers can improve and continue, or discontinue the interventions. The evaluation committee provided the collected information to all project managers who in turn streamlined, redesigned or abandoned the interventions.

Based on process evaluation results, IHHP interventions fall into 3 categories:

• Interventions that can be integrated into the cooperating organizations without carrying additional burdens in terms of financial or human resources. These were generally interventions designed according to organizational policies, priorities and rules.

• Interventions than can be integrated into the cooperating organizations providing that they are modified and aligned with the overall goals of the respective organizations. These interventions were confronted with financial difficulties or shortage of other resources because they were not in line with organizational goals. Such interventions were redesigned based on process evaluation results.

• Interventions that failed and could not be integrated into their target organizations in spite of modifications. Such interventions were eliminated as "unsuccessful and impractical". The latter interventions remain listed, as they may harbor the potential to be modified and improved for use in other areas. This, however, requires understanding the factors leading to their failure as reported in process evaluation results.

PE normally relies more on interviews with stakeholders and fidelity assessment, and to a lesser extent on observation [15]. In this study, factories constituted the venues of the Worksite Intervention Project. Factory workers are normally assigned to one of two or three working shifts. Based on the results, 39% of workers attended IHHP educational classes. This suggests that only daytime personnel were involved in the interventions. The subjects expressed satisfaction with the education they received and their performance with regard to nutrition and physical activity changed in 85% of cases. Hence, it can be argued that education is an effective measure for achieving lifestyle improvement in worksites. Given that every participant was exposed to a combination of educational methods, i.e., face-to-face education, class education and educational materials, no judgment can be made as to the most effective method of education. Education was delivered to factory workers through public health officers and physicians working at the factories who possessed more intimate knowledge of the conditions and needs of the personnel.

One of the factors contributing to the success of interventions is their congruence with quality promotion efforts made by factory managers. Indeed, the interventions can be integrated into the management's quality promotion programs and the existing financial and human resources can be employed to prevent additional burdens on the system. With the factory managers' endorsement and owing to increased budget and extension of exercise hours compared to before interventions, the workers' physical activity increased slightly with education. Moreover, the factory managers were satisfied with the strategy of interventions, since improvement of the workers' physical and mental health is bound to increased productivity.

Only 57% of the factories could improve their food menus within their existing means. These improvements included substituting hydrogenated fat with liquid oil, reducing red meat consumption and increasing the consumption of fish and vegetables. Nearly 50% of workers expressed satisfaction with the type and taste of healthy foods in their factory restaurant. On this note, mention can be made of the remarkable success of one of the factories in establishing a "Healthy Heart Restaurant". The restaurant, originally built to offer meals to 70 out of the 7000 workers, and has been expanded to cater for 1200, is currently unable to meet all demand for healthy food due to inadequate personnel, equipment and space.

Although non-smoking regulations are enforced in nearly 71% of factories, the number of smokers has not changed. IHHP impact studies have shown that although the community is adequately aware of the harms of smoking, the prevalence of smoking has remained unaltered in spite of educational and legislative interventions. This may be explained by the physically and psychologically addictive nature of smoking [16]. As education and increased awareness alone are unlikely to lead to smoking cessation, the individual's decision-making ability should be increased and appropriate smoking cessation methods employed to counter the physical and mental effects of smoking.

Apparently, environmental changes related to nutrition are easier to bring about than changes related to other risk factors. This probably indicates that the community's current attitude towards modifying nutritional habits is far better than its attitude to physical activity and smoking. Making changes related to physical activity and smoking requires further interventions aimed at education and improvement of attitudes, as well as appropriate environmental legislations and capacity building [17].

Our findings are in line with some studies conducted in workplaces in showing the feasibility of implementing interventions with simultaneous evaluation in workplaces [18,19]. As documented by a study in Maine, worksites are an ideal setting for interventions. Using community-based participatory research methodology increases community capacity for evaluation, dissemination, and use of evaluation results [18]. Worthy of noting is that many factories in Iran pay for the screening and treatment costs of their personnel. The managers have realized that improving lifestyle is an effective way to reduce such costs in the long run. Another important finding is the mangers willingness to cooperate with the program. Presently, interventions are implemented at factories where the managements' attitudes towards the program are acceptable. In some instances, change in a factory's management led to the separation of factory from the program. This has also been demonstrated in a study conducted by Lorig [20]. Another factor contributing to success in this program has been the regular contacts of project mangers with factory managers and public health officers. Inadequate communication was initially identified as one of the hurdles to the progress of the program. This problem was later addressed and overcome by project managers. Dissemination of the program requires regular contacts between the health system and worksites. The health system should monitor the implementation of interventions and provide appropriate feedback to factory managers, so that interventions can be gradually integrated into factories routines. The shortage of public health professionals in our community may be regarded as one of the obstacles to the dissemination of the program.

## Study limitations

Interventions of the type discussed in this study and their evaluation are very new in developing countries. Hence, they are tempered with several defects. Among the shortcomings affecting the program in discussion, reference can be made to the late start of PE. Evaluation data were partly collected using checklists from the beginning, however, systematic data collection and qualitative evaluation started with delay. Another limitation concerns the scale used in scoring the questionnaires. Several studies have reported using five-point and seven-point scoring scales. In this study, however, a three-point scoring scale was used which may not have been reflective of the entire spectrum of opinions. Despite all challenges and shortcomings, the PE is continuing in IHHP with a larger sample size and the use of a five-point scoring scale.

## Conclusion

The aims of interventions should be matched with the factory's organizational goals of interventions. Their benefits should be explained to the factory managers as well. Project managers should remain in regular contacts with factory managers and public health officers to keep them up-to-date with the results of interventions. Dissemination and success of such interventions in worksites depend heavily on the country's global health policies and the health system's priorities. In the current study, success was mainly shaped by the organizational readiness and timing of the implementation. Integrating most of the activities of the project to the existing routine activities of public health officers in worksites is suggested to be the most effective means of implementation of the health promoting activities in work places. The results of our experience may help other developing countries to plan for similar interventions.

## Competing interests

The authors declare that they have no competing interests.

## Authors' contributions

KR participated in the design of the study, performed the statistical analysis, participated in the sequence alignment and drafted the manuscript, RK participated in the design of the study and drafted the manuscript, NS participated in the design of the study, participated in the sequence alignment and drafted the manuscript, HA and MA participated in the design of the study, performed the statistical analysis and participated in the sequence alignment, KH, AB, MB, KZ and SS participated in the sequence alignment. All authors read and approved the final manuscript.

## Pre-publication history

The pre-publication history for this paper can be accessed here:


